# Hafnium-Based Ferroelectric Diodes for Logic-in-Memory Application

**DOI:** 10.3390/mi17010108

**Published:** 2026-01-14

**Authors:** Shuo Han, Yefan Zhang, Xi Wang, Peiwen Tong, Chuanzhi Liu, Qimiao Zeng, Jindong Liu, Xiao Huang, Qingjiang Li, Rongrong Cao, Wei Wang

**Affiliations:** College of Electronic Science and Technology, National University of Defense Technology, Changsha 410073, China; hanshuo19@nudt.edu.cn (S.H.); zhangyefan18@nudt.edu.cn (Y.Z.); wangxi@nudt.edu.cn (X.W.); tongpeiwen20@nudt.edu.cn (P.T.); liuchuanzhi@nudt.edu.cn (C.L.); zqm0815@email.swu.edu.cn (Q.Z.); liujindong24@nudt.edu.cn (J.L.); hx20011231hnly@163.com (X.H.); qingjiangli@nudt.edu.cn (Q.L.)

**Keywords:** ferroelectric diodes, bidirectional rectification, logic-in-memory devices

## Abstract

Due to the Von Neumann bottleneck of traditional CMOS computing, there is an urgent need to develop in-memory logic devices with low power consumption. In this work, we demonstrate ferroelectric diode devices based on the TiN/Hf_0.5_Zr_0.5_O_2_/HfO_2_/TiN structure, implementing 16 Boolean logic operations through single-step or multi-step (2–3 steps) cascade and achieving attojoule-level one-bit full-adder computation. The TiN/Hf_0.5_Zr_0.5_O_2_/HfO_2_/TiN ferroelectric diode exhibits non-destructive readout and bidirectional rectification characteristics, with the conduction mechanism following Schottky emission behavior in the on-state. Based on its bidirectional rectification characteristics, we designed and simulated the circuit scheme of 16 Boolean logic and one-bit full-adder through cascaded operations. Both the input and output logic values are represented in the form of resistance, without the need for additional form conversion circuits. The state writing is performed by pulse-controlled polarization flipping, and the state reading is non-destructive. The logic circuits in this work demonstrate superior performance with ultralow computing power consumption in simulation. This breakthrough establishes a foundation for developing energy-efficient and scalable in-memory computing systems.

## 1. Introduction

The Von Neumann architecture, while foundational in modern computing, suffers from inherent limitations due to the physical separation between the memory and processing units. This architectural constraint leads to the well-documented Von Neumann bottleneck, manifesting as significant energy overhead and throughput limitations during data-intensive operations [1,2,3,4,5]. Logic-in-Memory (LiM) architectures have emerged as a promising solution to circumvent this bottleneck by collocating computation and storage functionalities [1,2]. However, the traditional CMOS-based LiM implementations face critical challenges including data loss and compromised storage density which are induced by the volatility and transistor-intensive circuit designs, respectively.

Among the various types of non-volatile memories, ferroelectric memories, such as ferroelectric random access memory (FeRAM), which implements non-volatile storage by switching the polarization state, are regarded as a very promising storage solution due to their low power consumption, high speed, high endurance, and good retention [6]. Meanwhile, conventional ferroelectric materials face CMOS incompatibility challenges [7,8,9] and thickness-dependent ferroelectricity degradation [10,11,12,13], which constrain storage density enhancement and practical applications [14]. Hafnium-based oxide ferroelectric materials, particularly Hf_0.5_Zr_0.5_O_2_ (HZO), offer a novel approach to overcoming these limitations of conventional ferroelectric materials. As a new ferroelectric material, HZO exhibits a remarkable stability of the polarization state that maintains consistency over massive read/write cycles, offering distinct advantages in LiM. It also exhibits good compatibility with CMOS processes and maintains stable ferroelectricity at remarkably ultrathin thickness (<10 nm) [15]. Moreover, HZO-based ferroelectric devices exhibit nanosecond-scale switching speeds and superior logic operation performance. These characteristics render HZO-based ferroelectric devices ideal for LiM computing applications.

The current research in the field of ferroelectric devices for LiM computing is predominantly centered on FeRAM and FeFET [16], but there are still some limitations. The LiM computing operation based on FeRAM is complex, requires a large capacitance area, and the reading process is destructive, which necessitates data rewriting [17,18]. Although FeFET supports non-destructive data reading and 3D stacking [19,20,21], it requires the incorporation of additional selector devices during array integration. Alternatively, two-terminal devices, such as the ferroelectric tunnel junction (FTJ) and the ferroelectric diode (Fe diode), with simple structures and non-destructive data reading, have great potential for high-density array integration. The FTJ device utilizes ferroelectric materials as the barrier layer to generate a substantial tunneling resistance (TER) effect, but it demands an additional selector device to mitigate sneaking currents due to its linear current–voltage (I–V) characteristics. In contrast, the Fe diode device, the resistance of which is controlled by the ferroelectric polarization-modulated Schottky barrier, exhibits intrinsic nonlinearity and self-rectifying properties that enable selector-free crossbar arrays and cascadable logic implementation [6].

In this work, TiN/Hf_0.5_Zr_0.5_O_2_/HfO_2_/TiN Fe diodes were fabricated and the carrier transport mechanism was systematically investigated. The resistance of Fe diodes is modulated by the ferroelectric polarization-induced Schottky barrier. Thus, the rectification direction of Fe diodes is switchable because the ferroelectric polarization can be switched by the electric field, demonstrating bidirectional rectification characteristics. The on-state currents under both positive and negative electric field directions are related to the Schottky model, indicating that the transport mechanism of these Fe diodes is Schottky emission (SE). Moreover, the Fe diodes demonstrate the capacity for stable bidirectional rectifying memory windows and non-destructive reading, providing a foundation for LiM applications. We designed a bidirectional rectification-based mapping scheme between resistance states and input/output logic values, which enables non-volatile data storage without the need for additional signal conversion modules. Building on the resistance state mapping scheme and bidirectional rectification characteristics, we implemented all 16 Boolean logic operations (including NOT, NAND, NOR, etc.), and on this basis, one-bit full-adder was successfully constructed by cascading, with the feasibility of these circuits verified via simulations. Notably, the logic circuit schemes implemented in this work achieve ultralow power consumption at the aJ scale. The bidirectional rectification characteristics of Fe diode devices facilitate the cascade expansion of logic gates and the array self-selection ability, providing significant technical support for high-density integration.

## 2. Experiments

Firstly, the Si/SiO_2_ substrate was pretreated and cleaned by ultrasonic cleaning with acetone, absolute ethanol, and deionized water. Then, it was dried with high-purity N_2_. Subsequently, a 20 nm TiN film was deposited on the substrate by magnetron sputtering. After deposition, the bottom electrode was patterned by a UV lithography process, and then an H_2_O_2_ solution was used to etch. After that, a 10 nm HZO film was deposited by atomic layer deposition (ALD). The deposition temperature of the ALD process was controlled at 280 °C, and the deposition cycle ratio of HfO_2_ to ZrO_2_ was maintained at 1:1. TEMAH, TEMAZ, and deionized water were used as precursors for Hf, Zr, and O, respectively. In the ALD process, the pulse duration of TEMAH and TEMAZ is 300 ms, and that of deionized water is 150 ms, with N_2_ purge for 4 s. After the deposition of HZO, the sample was annealed in a rapid thermal annealing (RTA) device under a N_2_ atmosphere at 500 °C for 30 s, with a heating rate of 50 °C/s. Next, an 8 nm HfO_2_ film was deposited as the dielectric interlayer via the ALD technique. Finally, a 20 nm TiN film was deposited using magnetron sputtering and subsequently patterned via a UV lithography process and the lift-off process.

## 3. Results and Discussion

**Figure 1:**
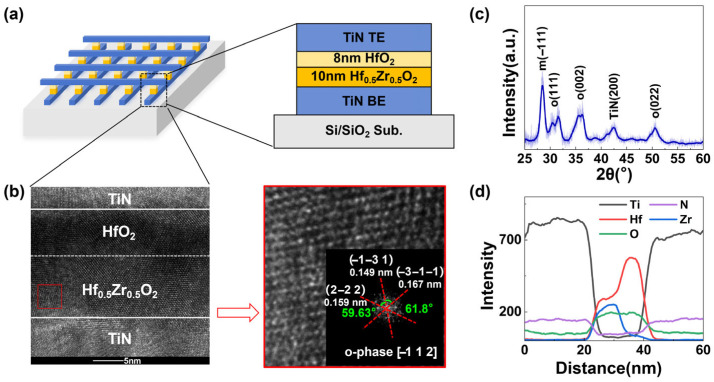
(**a**) A schematic view of Fe diode array and diagram of Fe diode structure. (**b**) HRTEM image of Fe diode with FFT pattern. (**c**) GIXRD patterns for 8 nm HfO_2_ and 10 nm HZO device. (**d**) EDS line-scan image of the Fe diode.

Figure 1a shows the schematic of the Fe diode array and the diagram of the TiN/HZO/HfO_2_/TiN structure is shown on the right side. As illustrated in Figure 1b, the interfaces of the layers are clearly visible in the high-resolution cross-sectional transmission electron microscopy (HRTEM) image of the device. The fast Fourier transform (FFT) pattern of the red boxed region is shown on the right, verifying the non-centrosymmetric orthorhombic phase (Pca2_1_) in the HZO film. At an incidence angle of 0.5°, o (111) and o (002) crystalline phase diffraction peaks are observed at 30.5° and 35.5° diffraction angles of the grazing-incidence X-ray diffraction (GIXRD) pattern of the sample in Figure 1c, indicating that the HZO film possesses ferroelectricity. Meanwhile, a peak also appears at 28.5°, which is due to the single-crystalline structure of the 8 nm HfO_2_ layer without heat treatment [22]. This result confirms the coexistence of the ferroelectric orthorhombic phase in HZO and the monoclinic phase in HfO_2_, where the HZO layer provides the ferroelectric function and the HfO_2_ intermediate layer acts as the dielectric barrier layer, thus corresponding to the design of the device stack structure. Figure 1d shows the results of the energy dispersive X-ray spectroscopy (EDS) line scans acquired along the vertical cross-sectional direction (from the lower electrode to the upper electrode). The element distribution profile clearly shows that the Ti and N signals are limited to the TiN electrode at the top and bottom without diffusion, indicating the integrity of the electrode layer, Hf and Zr signals are only limited to the HZO layer, confirming the uniform doping of Hf/Zr in the ferroelectric layer, and O signals are uniformly distributed in the HfO_2_ and HZO oxide layer without oxygen segregation or vacancies. It fully verifies the structural integrity of the design for dependable in-memory computation operations.

**Figure 2:**
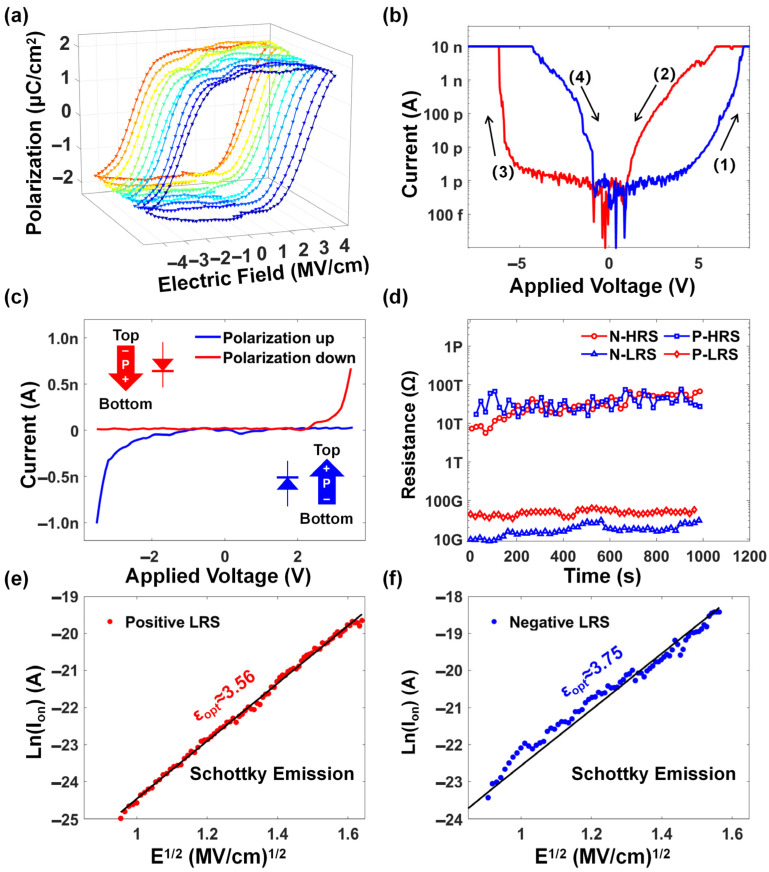
(**a**) Ten consecutive P–E loops of Fe diode. (**b**) I–V characteristics curve of the device. (**c**) Bidirectional rectification characteristics of the Fe diode with a corresponding schematic of ferroelectric polarization. (**d**) Retention characteristics of the Fe diode device’s 4 resistance states. Current fitting of the LRS under (**e**) positive voltage sweep and (**f**) negative voltage sweep.

Figure 2a shows the 10 consecutive polarization–electric field (P–E) curves of the Fe diode. The ferroelectric response was characterized by a positive-up-negative-down (PUND) measurement, which we prefer to avoid the influence of leakage current effects [23], and it can be observed that the device exhibits stable ferroelectricity. The I–V characteristics of the Fe diode are shown in Figure 2b, and the device shows a distinct memory window. The coercive field of the P–E curve is close to the transition voltage of the I–V curve, which indicates that the polarization reversal of the ferroelectric material in the device plays a regulatory role. The I–V scan sequence is 0 V → 8 V → 0 V → −8 V → 0 V, corresponding to the four sequential processes (1)–(4), marked in Figure 2b. The red and blue curves in Figure 2b represent the forward and reverse rectification curves, respectively, corresponding to the forward and reverse polarization of the Fe diode.

When a positive scanning voltage was applied to the device, the polarization direction of the device is downward; the resistance state undergoes a transition from the high resistance state (HRS) to the low resistance state (LRS) and then exhibits diode characteristics with rectification behavior under a low positive reading voltage. When a negative scanning voltage is applied, the polarization and rectification direction then switch, and the bidirectional rectification characteristic curve shown in Figure 2c is obtained. The device displays switchable diode characteristics, indicating that the resistance state is adjustable. Furthermore, the resistive states of the device can be divided into four types according to different rectification directions: low resistive state under positive bias (P-LRS), high resistive state under negative bias (N-HRS), high resistive state under positive bias (P-HRS), and low resistive state under negative bias (N-LRS). To evaluate the retention characteristics of the Fe diode with bidirectional rectification behavior, the device is first written to the corresponding LRS by positive or negative DC scanning and then read by both positive and negative voltage at 2.5 V. Figure 2d shows that all four resistance states exhibit good retention performance exceeding 1000 s, which provides a guarantee for the application in LiM.

It has been demonstrated in other work that the transmission model of devices is complicated due to the presence of different structures and preparation processes [22,24,25,26]. For instance, HZO ferroelectric devices, including ferroelectric capacitors, ferroelectric transistors (FeFETs), and ferroelectric tunnel junctions (FTJs), have been observed to exhibit behavior consistent with the Schottky model [27], the Poole–Frenkel model [28], or the tunneling model [29]. Given that the Fe diode in this work possessed the characteristics of programmable and bidirectional operation, which differ from those of other HZO devices, it is imperative to conduct a comprehensive study on the physical mechanism of electron transport. Figure 2e,f redraws the curve of the on-state current (I_on_) with the electric field (E) in both the positive and negative rectification states, represented by Ln (I_on_)–E^0.5^. We performed a linear fit on the curve of the non-limiting region, and the results showed linear behavior under both P-LRS and N-LRS conditions, indicating that the on-state current is related to the Schottky emission (SE) current. This relationship aligns with the Richardson–Schottky equation [6,24]:

(1) $$I=AA^{*}T^{2}exp\left[\frac{-q\left(\emptyset_{B}-\sqrt{qE/4\pi \varepsilon_{0}\varepsilon_{opt}}\right)}{k_{0}T}\right]$$

where A is the device area, $A^{*}$ is the effective Richardson constant, *T* is the Kelvin temperature, q is the elementary charge, $\varepsilon_{0}$ is the vacuum permittivity, $\varepsilon_{\mathrm{opt}}$ is the optical permittivity of HZO films, $k_{0}$ is the Boltzmann constant, and $\emptyset_{B}$ is the Schottky barrier energy of the metal–insulator interface.

The $\varepsilon_{\mathrm{opt}}\;$ extracted from the slope of the linear fitting line is around 3–4, which is close to the theoretically calculated HZO optical dielectric constant (~4). In addition, the $\emptyset_{B}$ value is obtained by the intercept of the linear fit, revealing that the $\emptyset_{B}$ value is ~1 eV at the HZO/BE interface. The work function of TiN and the electron affinity of HZO are ~4.7 eV and ~2.7 eV, respectively, which means that the initial $\emptyset_{B}$ without a polarization contribution is 2 eV. However, the actual $\emptyset_{B}$ should be less than 2 eV due to the downward polarization state and the presence of a bottom electrode oxidation interface layer [26]. Therefore, the extracted value of 1 eV is reasonable and indicates that the SE mechanism can effectively evaluate current changes. Furthermore, a fitting of Poole–Frenkel emission was conducted since it also exhibited a similar linear fit. However, the corresponding extracted values are significantly different from the theoretical values, indicating that the conductive mechanism of the Fe diode is dominated by the Schottky model rather than the Poole–Frenkel model [26,30,31].

From the perspective of the energy band, here is an explanation for the principle of the transport mechanism in the LRS of the device [22,24]. After positive ferroelectric polarization, the energy band of the HZO layer bends relative to the polarization direction. The conduction band energy level at the polarization-direction side (TE) was high, which formed an electron barrier that hindered electron injection. Meanwhile, the positive charge of the polarization on the other side (BE) was attracted, and electrons spontaneously gathered on the BE side. When the applied voltage was consistent with the polarization direction, the positive voltage weakened the barrier height and allowed electrons to rapidly inject the BE side, forming the LRS. Consequently, a substantial Fe diode current was generated, which aligns with the Schottky model. When the applied voltage was opposite to the polarization direction, the increased barrier height suppressed the sneaking current and enhanced the barrier isolation effect due to the amorphous structure of the HfO_2_ layer. Due to the higher barrier to overcome, the injection of electrons poses a significant challenge leading to an exceedingly small conduction current, which effectively maintains the contrast and retention characteristics of the high and low resistance states of the device [22].

Based on the verified conduction mechanism of the LRS, we developed the simulation model of Fe diodes to implement Boolean logic operations. In this work, we presented circuit schemes based on the Fe diode for 16 Boolean logic, where the input and output data were stored in the device in the form of resistance and calculated by different circuit combinations. As mentioned above, the rectification direction of the device is divided into two types. LRS under a certain bias also implies HRS under reverse bias. Therefore, for the uniqueness of the logical value definition, the read voltage direction is defined as positive voltage applied on the TE and ground connection to the BE. In this case, the device’s P-HRS is designated as logic state “0” and P-LRS as logic state “1”, a definition that underpins all subsequent logic circuit designs and operational implementations. Different states can be written to the device by pulse-controlled polarization switching in different directions, and non-destructive reading is achieved by lower bias voltages.

**Figure 3:**
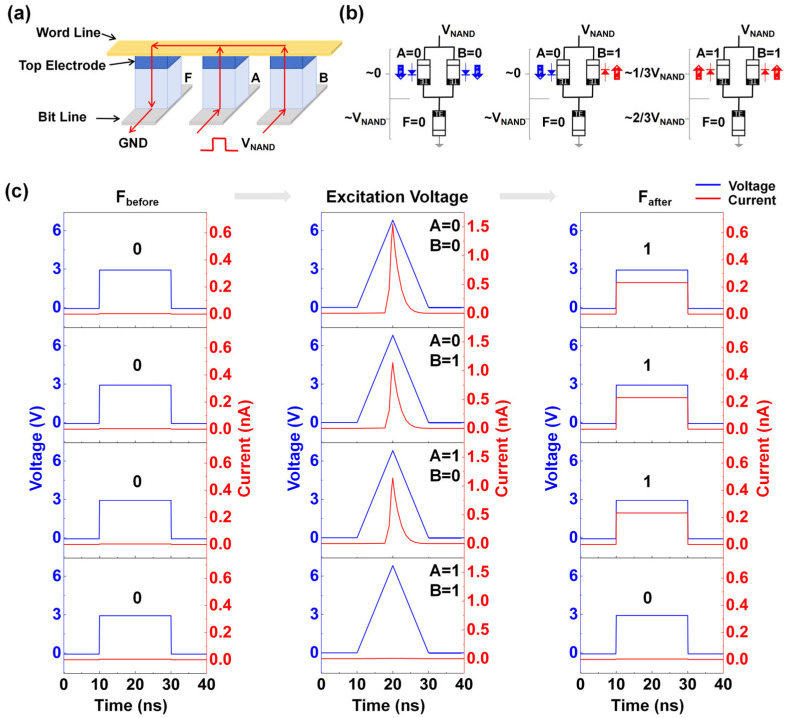
(**a**) Schematic diagram of the Fe diode-based NAND logic circuit scheme. (**b**) Equivalent circuit diagrams of the NAND logic circuit under 4 input combinations. (**c**) Voltage and current waveform diagrams of the circuit under 4 input combinations.

Then, we designed and implemented a circuit scheme for realizing the logic function F = A NAND B (where A and B are input variables, and F is the output variable), as shown in Figure 3a. Figure 3b illustrates the equivalent circuit diagram of the NAND circuit along with the polarization states of the devices. The initial state of the device F is set to 0, and the A·B are set to 0·0, 0·1/1·0, and 1·1, respectively. The voltage across device F varies with different combinations of logic value inputs. Specifically, since devices A and B exhibit different resistance states in the circuit under different inputs, the total resistance of their parallel structures will also change. Then, the voltage across device F in the circuit will change accordingly: the parallel resistance is smaller and the voltage drop on the parallel structure is smaller, thereby increasing the voltage of F. On the contrary, when the parallel resistance is larger, the voltage of F will be lower. Consequently, when the voltage across device F exceeds the transition voltage, the output of device F is written to state 1. A lower voltage does not alter the original state, and the state of F remains at 0. Figure 3c illustrates the diagram of excitation voltage and current change in the circuit and shows the output values of device F under four inputs: A·B = 0·0, 0·1, 1·0, and 1·1, which verifies the implementation of the NAND logic circuit scheme.

Table 1 presents a comprehensive overview of the corresponding circuit structures and implementations of all 16 Boolean logic. A and B are input devices, and X functions as the device that stores the intermediate value or the final output value F. In general, ten logic operations such as TRUE, NOT, NAND, NOR, etc., can be implemented in one step. As illustrated in the table, other logic operations such as AND, OR, etc., can be realized in two or three steps, and the excitation voltage of each device varies with time, corresponding to the different steps. Concurrently, to reduce the number of devices required for single-step operation, the output values of the four logic operations of IMP, NIMP, RIMP, and RNIMP will be stored in the input device, which will result in a partial change in the input state. Through simulation, the above 16 Boolean logic schemes based on Fe diodes possess ultralow power consumption as low as aJ level.

Then, we implemented a one-bit full-adder by cascading Fe diode-based logic gates. One-bit full-adder is the core unit for implementing binary addition. It adds two inputs (A and B) with the lower carry (C) and outputs the sum result (S) and the new carry (C_i+1_). Since the calculation processes of S and C_i+1_ are similar, we simplify the description by taking the calculation of S as an example, whose logical expression can be decomposed into the following:

(2) $$S=A\;⨁\;B\;⨁\;C=\bar{\left(A+B+\bar{C})\cdot (A+\bar{B}+C)\cdot (\bar{A}+B+C)\cdot (\bar{A}+\bar{B}+\bar{C}\right)}$$

Since the NOT and NAND logic gates based on Fe diodes can be realized in one step, the scheme of the output S of one-bit full-adder in this work is realized by cascading these two logic gates. Let F1, F2, F3, and F4 be the intermediate values of the operation process, and we can obtain the following:

(3) $$\begin{array}{c} S=A\;⨁\;B\;⨁\;C=\bar{F1\cdot F2\cdot F3\cdot F4}\\ F1=A+B+\bar{C}=\bar{\bar{A}\cdot \bar{B}\cdot C}\\ F2=A+\bar{B}+C=\bar{\bar{A}\cdot B\cdot \bar{C}}\\ F3=\bar{A}+B+C=\bar{A\cdot \bar{B}\cdot \bar{C}}\\ F4=\bar{A}+\bar{B}+\bar{C}=\bar{A\cdot B\cdot C} \end{array}$$

**Figure 4:**
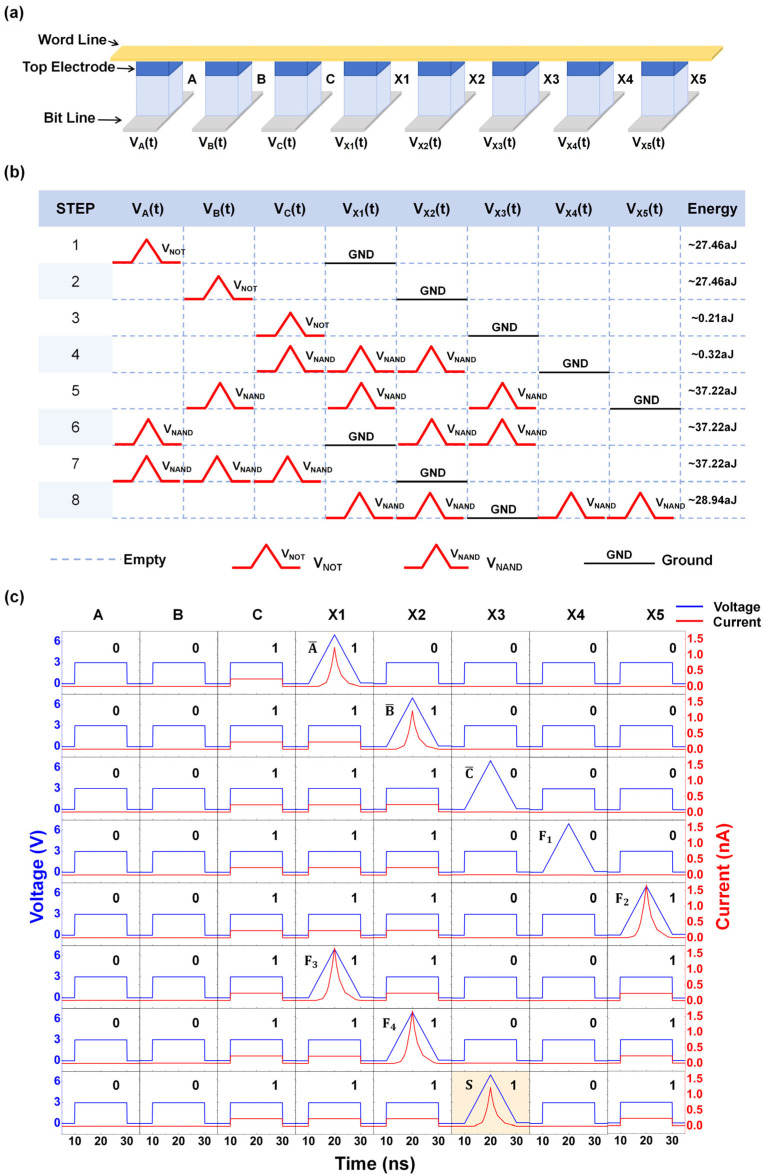
(**a**) Circuit schematic of the one-bit full-adder. (**b**) Timing diagram of voltage excitation application (pulse width = 20 ns) and power consumption under input combination A·B·C = 0·0·1. (**c**) Device data variation diagram during each operation step under input combination A·B·C = 0·0·1.

We present the schematic diagram of the one-bit full-adder circuit in Figure 4a, which requires only eight devices and eight operation steps to obtain the output value S. This circuit includes three input devices and five devices for storing intermediate values. The voltage timing diagram of the circuit for each step of the operation is shown in Figure 4b. In the first three steps, we implemented NOT operation for A, B, and C, and the results were stored in the devices X1, X2, and X3, respectively. In step 4, a NAND operation was performed with $\bar{A}$, $\bar{B,}$ and C as inputs, and the intermediate value F1 was stored in device X4. Similarly, the value of F2 was stored in X5 in step 5. Since some of the intermediate values would not be involved in subsequent operations, the value of F3 was rewritten into X1 at step 6 and the value of F4 was stored in X2 in step 7. The result S was written in the last step and stored in device X3, obtained by performing a NAND operation with the four intermediate values.

Figure 4c shows the images of data changes in the devices that are being written in each step in the case of the input A·B·C = 0·0·1, as well as the rest of the unwritten devices’ state diagrams. In the first two steps, devices X1 and X2 were written with 1, which corresponds to the value of $\bar{A}$ and $\bar{B}$ respectively, and in the third step, device X3 was written with 0 correspondingly. In steps 4 to 7, the four intermediate values were solved by NAND operation, obtaining F1 = 0, F2 = 1, F3 = 1, and F4 = 1, respectively, which is consistent with the results of the logic operations. Finally, these four values were subjected to a NAND operation and computed to obtain that S = 1. The result was stored in X3, which verified the feasibility of the above scheme. Meanwhile, the power consumption of each step when inputting A·B·C = 0·0·1 is displayed in the last column of Figure 4b. This circuit completely inherits the ultralow power consumption characteristics of the Fe diode-based 16 Boolean logic listed in Table 1, and the total power consumption of the eight-step operation to calculate the value of S is only 196.05 aJ under the input A·B·C = 0·0·1.

As shown in Table 2, the Fe diode-based logic scheme in this work has a simple structure without any additional devices. This advantage derives from the bidirectional rectification of Fe diodes, which enables the direct mapping of the resistance state-to-logic value. The scheme also features non-destructive read, implements all 16 Boolean logic operations, and realizes a one-bit full-adder through cascading. The power consumption of A NAND B is only 17.07 aJ, and the average power consumption of all 16 Boolean logic is as low as 88.17 aJ. Meanwhile, the power consumption of the one-bit full-adder computing S and C_i+1_ under eight different inputs are 178.22 aJ and 67.91 aJ, respectively, and the total power consumption is as low as 246.13 aJ. It should be noted that although the total power consumption of the one-bit full-adder is higher than that of a single NAND gate, this difference is determined by the cascade operations. The total power consumption of the one-bit full-adder is the accumulation of multi-step operations. However, when focusing on per-step power consumption, the average single operation power consumption of the one-bit full-adder is approximately 20.51 aJ, which is at the same low-power level as the 17.07 aJ of the two-input NAND gate. Therefore, even when implementing complex full-adder functions, the logic scheme in this work can complete cascaded operations with controllable total power consumption. Compared with other works, our scheme has significant advantages in the number of required devices, additional circuit overhead, and computing power consumption, providing core technical support for the next generation of low-power LiM applications [32,33,34,35].

## 4. Conclusions

In this work, we constructed a TiN/Hf_0.5_Zr_0.5_O_2_/HfO_2_/TiN Fe diode device, which exhibits switchable bidirectional rectification behavior and good memory reliability. The transport mechanism was investigated through a series of electrical tests and mathematical fitting for optimizing the device performance, indicating that the carrier transport in the on-state mainly follows the Schottky model. We proposed 16 Boolean logic operation schemes based on the bidirectional rectification behavior of Fe diodes and realized a one-bit full-adder application by cascading. The Fe diode-based logic scheme in this work stores data in the form of device resistance and performs the operation through circuit combinations without additional conversions. It outperforms other devices by offering a fab-friendly structure and ultralow computing power consumption (aJ-scale), which provides a new path for LiM.

## Figures and Tables

**Table 1:** 16 Boolean logic implementation circuit schemes based on Fe diodes.

Logic Operation	Number of Devices	Number of Steps	Functions	Circuit Structures	Input State Preservation
TRUE, FALSE	1	1	1, 0	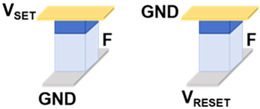	Yes
NOT A, NOT B	2	1	$\bar{A},\;\bar{B}$	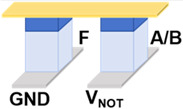	Yes
NAND, NOR	3	1	$\bar{AB},\;\bar{A+B}$	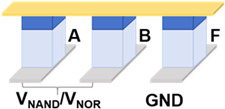	Yes
IMP, NIMP, NIMP, RIMP	2	1	$\bar{A}+B,\;A\bar{B},$ $\bar{A}B,\;A+\bar{B}$	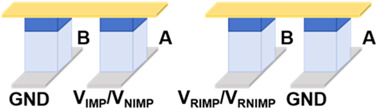	Partially Change
COPY A, COPY B	3	2	$A=\overset{=}{A},\;B\overset{=}{B}$	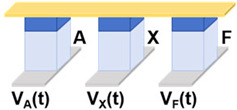	Yes
AND, OR	4	2	$AB=\bar{\bar{AB}},$ $A+B=\bar{\bar{A+B}}$	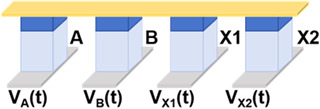	Yes
XNOR	4	3	$AB+\bar{AB}=\bar{A+B}+AB=\bar{A+B}+\bar{\bar{AB}}$	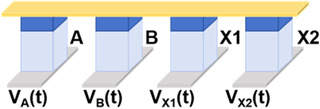	Partially Change
XOR	5	3	$A\bar{B}+\bar{A}B=\bar{\left(A+\bar{B})\cdot (\bar{A}+B\right)}$	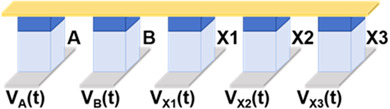	Partially Change

**Table 2:** Performance comparison of LiM devices.

Cell Structure for LiM Device	Double Gate Charge-Trapping Memory (Al_2_O_3_ as Blocking Oxide) [32]	Memoristor (Ti/HfSe_x_O_y_/ HfSe_2_/Au) [33]	FTJ (TiN/HZO/Al_2_O_3_/ TiAlN) [34]	FeFET (TiN/Si:HfO_2_/ SiON/Si) [35]	This Work
Additional Device Required	No	No	Yes (with nFETs)	Yes (with FETs)	No
Destructive Read	No	No	No	No	No
Boolean Logic Functions Realized	16	3 (XOR, IMP, NAND)	1 (XOR)	NA	16
One-bit Full-adder Realized	No	No	Yes	Yes	Yes
Power Consumption of A NAND B	~22.5 fJ	~0.3 fJ	NA	NA	17.07 aJ
Power Consumption of all 16 Boolean Logic	18–25 fJ	0.1 fJ–0.1 pJ	NA	NA	88.17 aJ (average)
Number of Devices in One-bit Full-Adder	NA	NA	3 FTJs and 15 FETs	7 nFETs, 7 FeFETs, and 6 FETs (in auxiliary circuit)	8 Fe diodes
Power Consumption of One-bit Full-Adder	NA	NA	12.3 fJ	15.9 fJ	246.13 aJ

The blue background is to emphasize the various features of this work.

## Data Availability

The data presented in this study are available on request from the corresponding author. (The data are not publicly available due to privacy).
